# Synthesis and crystal structure of sodium (ethane-1,2-di­yl)bis­[(3-meth­oxy­prop­yl)phosphinodi­thiol­ate] octa­hydrate

**DOI:** 10.1107/S2056989024009642

**Published:** 2024-10-08

**Authors:** Bryan P. Nell, David R. Tyler, Lev N. Zakharov, Dean H. Johnston

**Affiliations:** a Department of Chemistry Ripon College,Ripon WI 54971 USA; b Department of Chemistry and Biochemistry University of Oregon,Eugene OR 97403 USA; c Department of Chemistry, Otterbein University, Westerville, OH 43081, USA; University of Missouri-Columbia, USA

**Keywords:** di­thio­phosphinate, phosphinodi­thiol­ate, crystal structure

## Abstract

The title compound, *catena*-poly[[tri­aqua­sodium]-di-μ-aqua-[tri­aqua­sodium]-μ-(ethane-1,2-di­yl)bis­[(3-meth­oxy­prop­yl)phosphinodi­thiol­ato]], [Na_2_(C_10_H_22_O_2_P_2_S_4_)(H_2_O)_8_]_*n*_, crystallizes in the triclinic space group *P*1. The dianionic [CH_3_O(CH_2_)_3_P(=S)(S—)CH_2_CH_2_P(=S)(S—)(CH_2_)_3_OCH_3_]^2−^ ligand fragments are joined by a dicationic [Na_2_(H_2_O)_8_]^2+^ cluster that includes the oxygen of the meth­oxy­propyl unit of the ligand to form infinite chains.

## Abstract 2

In the title compound, the dianionic [CH_3_O(CH_2_)_3_P(=S)(S—)CH_2_CH_2_P(=S)(S—)(CH_2_)_3_OCH_3_]^2−^ ligand fragments are joined by a dicationic [Na_2_(H_2_O)_8_]^2+^ cluster that includes the oxygen of the meth­oxy­propyl unit of the ligand to form infinite chains.

## 1. Chemical context

Complexes of the type Fe(P_2_)_2_*X*_2_ have been shown to react with di­nitro­gen at high pressure to form [Fe(P_2_)_2_(N_2_)*X*]^+^ (Miller *et al.*, 2002 ▸). This reaction can potentially be used to scrub di­nitro­gen-contaminated natural gas. Unfortunately, the phosphine ligands in these di­nitro­gen-scrubbing complexes slowly dissociate in aqueous solution leading to degradation of the complexes, preventing a practical pressure-swing process from being developed. One potential method to develop complexes that are more robust is to use a phosphine macrocycle in place of the two bidentate ligands. For background on phosphine macrocycles, see: Caminade & Majoral (1994 ▸); Swor & Tyler (2011 ▸). The ‘macrocycle effect’ predicts that the binding constant for a macrocyclic ligand is orders of magnitude higher than the binding constant for two bidentate ligands (Melson, 1979 ▸).

In addition to their usefulness in the N_2_-scrubbing scheme described above, macrocyclic phosphine compounds are sought after in general as ligands for transition-metal complexes because of their strong binding properties. However, the synthesis of phosphine macrocycles is a relatively underdeveloped area. One approach to macrocyclic phosphines is a template synthesis in which two secondary bidentate phosphines are coordinated to a common metal center and then covalently linked (Lambert & Desreux, 2000 ▸; Nell & Tyler, 2014 ▸). Previously, we showed that complexes of the [Cu(P_2_)_2_]^+^ type (where P_2_ is a bidentate secondary phosphine) can react under basic conditions with various dihalides to form macrocyclic tetra­phosphine Cu complexes (Nell *et al.*, 2016 ▸). The title mol­ecule is an unexpected side-product of the process of removing the Cu metal center using aqueous NaSH to react with the Cu metal (Costantino *et al.*, 2008 ▸). Inter­estingly in this case, the P_2_ ligand was 1,2-bis­(meth­oxy­prop­yl)phosphino­ethane (MeOPrPE), which has been oxidized to the di­thio­phosphinate species, a reaction commonly encountered between secondary phosphines and elemental sulfur.
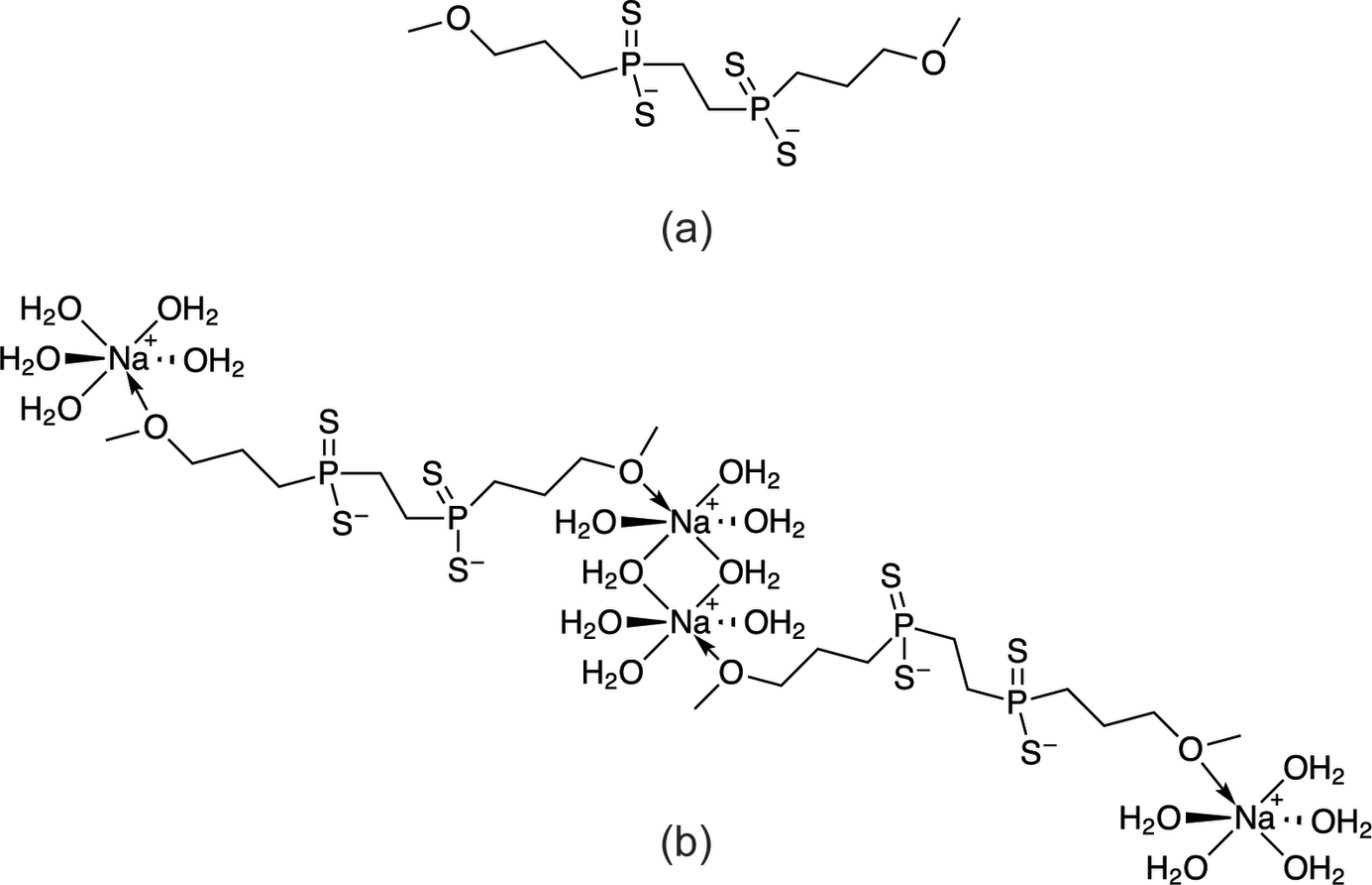


## 2. Structural commentary

**Figure 1:**
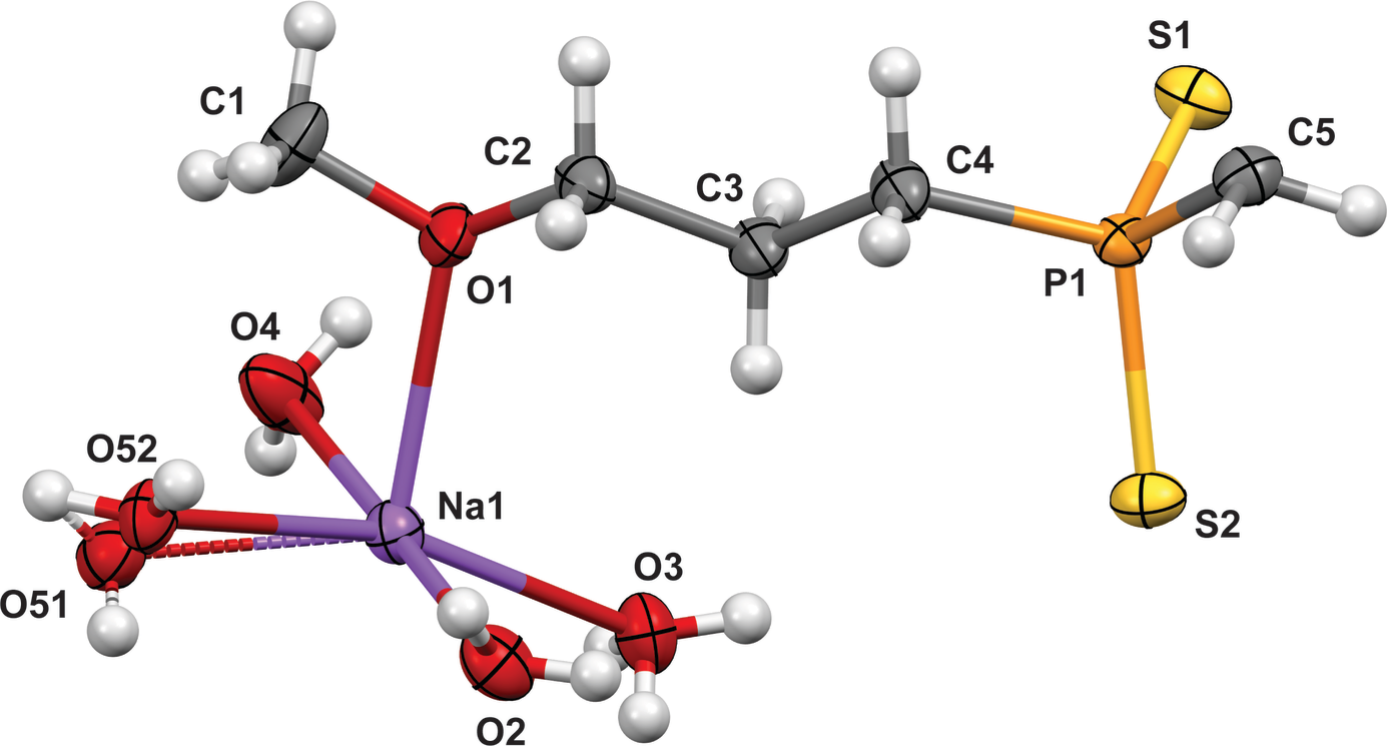
Displacement ellipsoid (50%) diagram and atom-numbering scheme for the asymmetric unit of the title compound.

The title compound is a P,S,Na-complex in which there are two P(=S)(—S) groups consisting of two terminal sulfur atoms bonded to each phospho­rus atom, providing a −2 charge. Two sodium cations and eight water mol­ecules form [Na_2_(H_2_O)_8_]^2+^ bridges between the anions. The oxygen of the meth­oxy­propyl unit of the ligand is also bonded to the [Na_2_(H_2_O)_8_]^2+^ cluster, completing a pseudo-octa­hedral coordination environment around each sodium cation and linking the cations and anions to form an infinite chain. The asymmetric unit (see Fig. 1 ▸) contains half of one dianionic bis­(phosphinodi­thiol­ate) chain, one sodium cation, and four water mol­ecules, one of which is disordered over two positions with 50:50 occupancy.

**Figure 2:**
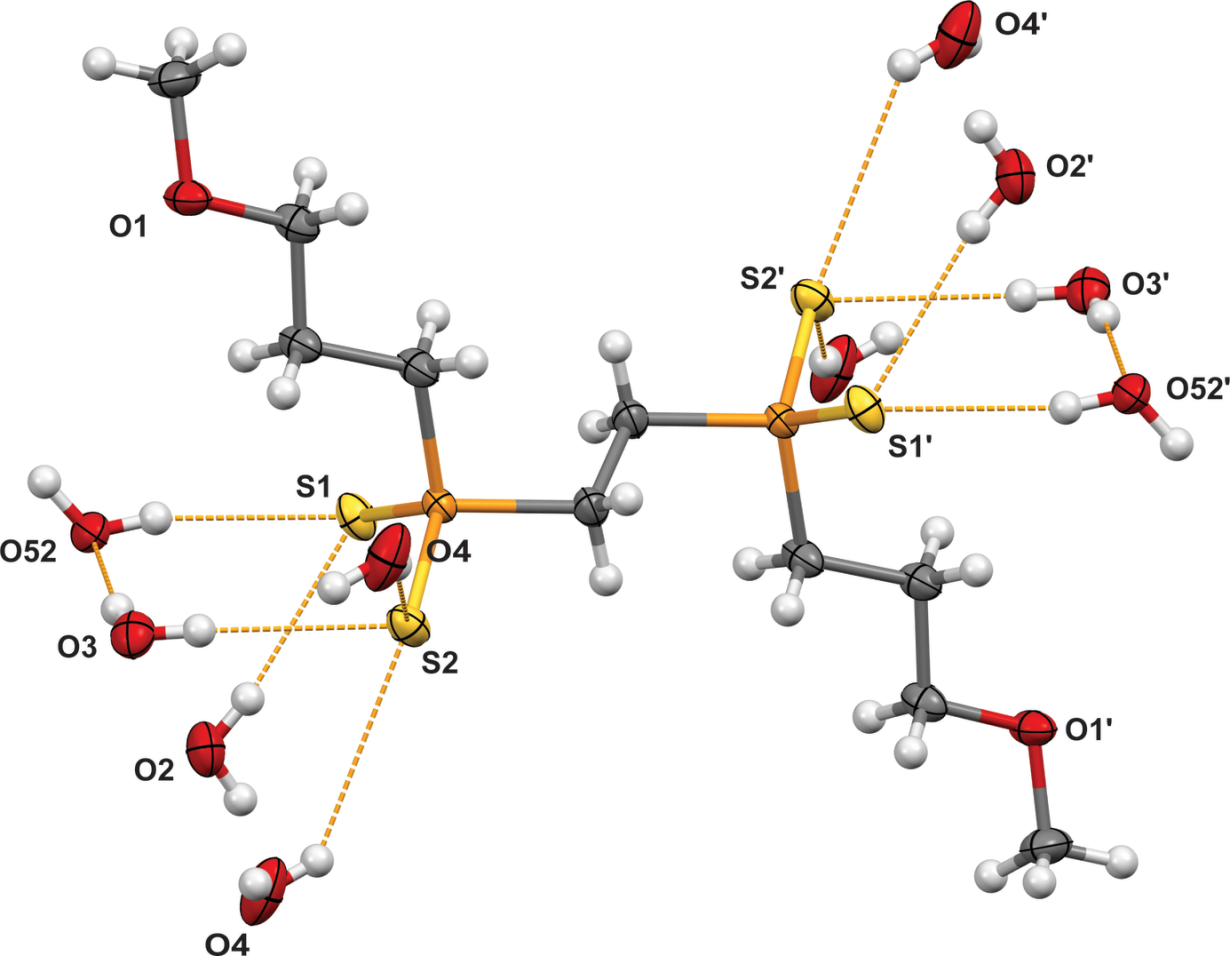
Displacement ellipsoid (50%) diagram showing O—H⋯S contacts in orange with selected atom labels. Only one position of the disordered water mol­ecule is shown for clarity. See Table 1 ▸ for donor–acceptor distances and angles.

Inter­estingly the lengths of the two P—S bonds for the phospho­rus atom P1 differ by 0.0206 (6) Å. While the structure can formally be described as having one phospho­rus–sulfur single bond and one double bond, clearly these should be equivalent by resonance. Indeed, comparison to seven similar di­alkyl­phosphinodi­thiol­ate structures (Pinkerton, 1990 ▸; Ebels *et al.*, 1997 ▸; Klevtsova *et al.*, 2003 ▸; Kokina *et al.*, 2008 ▸, 2010 ▸; Marc *et al.*, 2012 ▸; Guo *et al.*, 2022 ▸) shows that the average difference in phospho­rus–sulfur bonds in these compounds is only 0.006 Å. A closer look at the hydrogen bonding in the structure shows that the asymmetry is likely due to the fact that sulfur S1 has two hydrogen-bonding contacts (see Fig. 2 ▸, Table 1 ▸) compared to the three contacts for S2. A similar asymmetry in phospho­rus–sulfur bond lengths is observed in the structure of sodium di­ethyl­dithio­phosphinate dihydrate (Svensson & Albertsson, 1989 ▸), though the hydrogen-bonding network is quite symmetrical in that structure.

**Figure 3:**
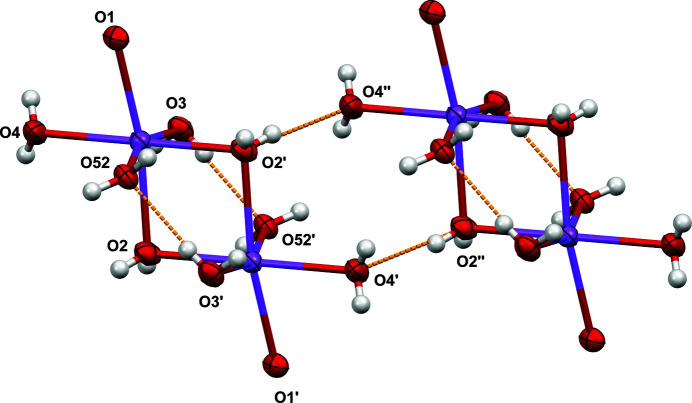
Displacement ellipsoid (50%) diagram showing O—H⋯O contacts in orange with selected atom labels. Only one position of the disordered water mol­ecule is shown for clarity. See Table 1 ▸ for donor–acceptor distances and angles.

The sodium ion and water mol­ecules form [Na_2_(H_2_O)_8_]^2+^ dimers around an inversion center. The coordination sphere of the sodium ion is filled by two bridging water mol­ecules (O2), three terminal water mol­ecules (O3, O4, and O51/O52), and one ether oxygen of the ligand mol­ecule (O1). As shown in Fig. 3 ▸, there is one intra-dimer hydrogen bond (O3—H3*C*⋯O52). A similar contact, O51—H51*A*⋯O3, is present in the second component of the disorder but is not shown in Fig. 3 ▸. The dimers are linked by additional hydrogen-bond contacts between atoms O2 and O4 of neighboring dimers (O2—H2*D*⋯O4). An additional intra­dimer contact, O4—H4*D*⋯O51, is present but not shown in Fig. 3 ▸.

## 3. Supra­molecular features

**Figure 4:**
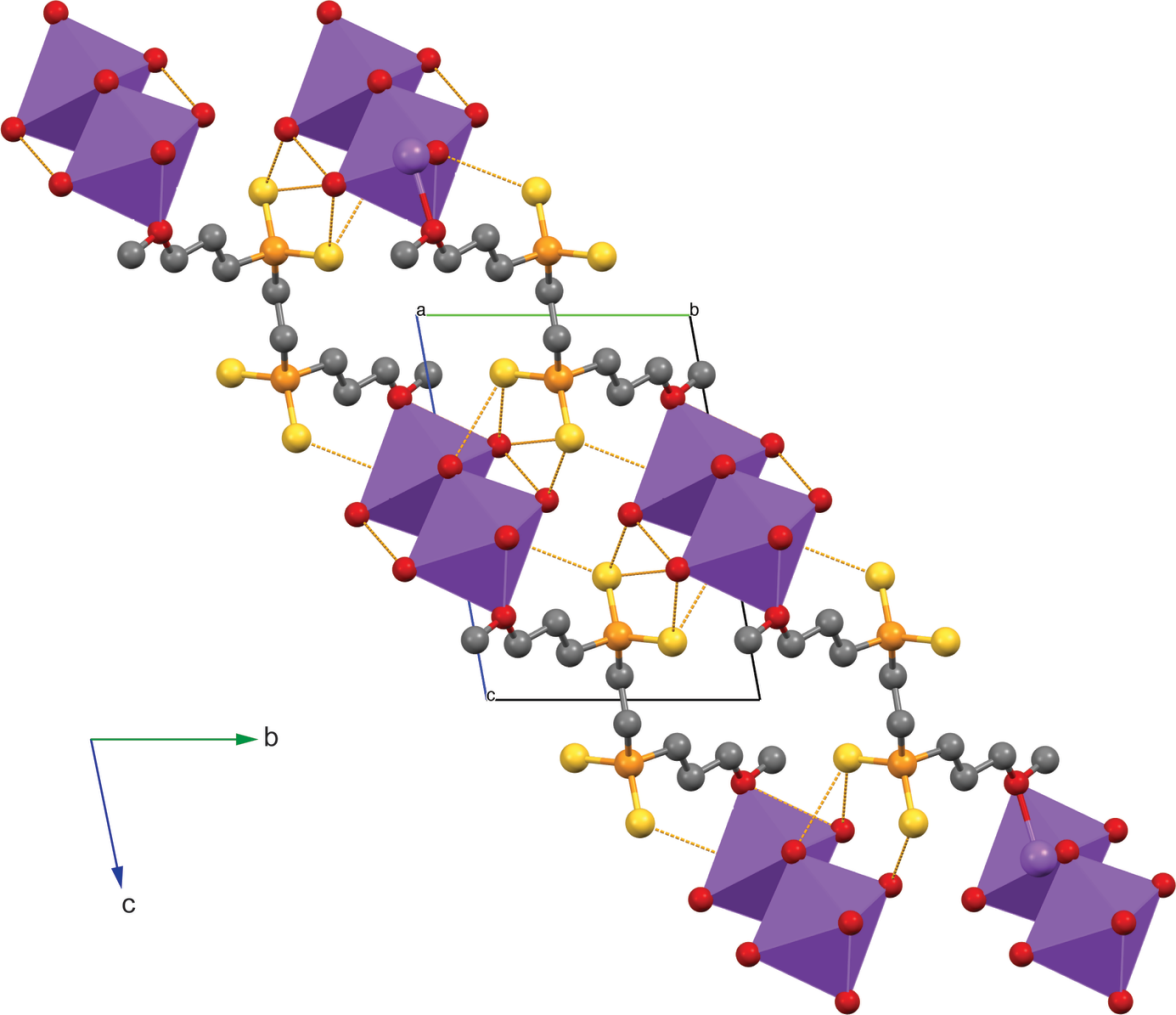
Polyhedral representation showing [Na_2_(H_2_O)_8_]^2+^ dimers (purple) linked by bis­(phosphinodi­thiol­ate) chains. The O—H⋯S contacts are shown in orange. Only one position of the disordered water mol­ecule is shown and hydrogen atoms omitted for clarity.

The sodium ion–water dimers can be visualized as edge-sharing octa­hedra (see Fig. 4 ▸) with two of the outer oxygen positions occupied by equivalent ether oxygen atoms from one end of the bis­(phosphinodi­thiol­ate) ligand. The ligands then link successive sodium ion dimers, forming infinite zigzag chains running parallel to the (0
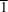
1) plane. The hydrogen-bonding inter­actions between the water mol­ecules and the sulfur atoms (Table 1 ▸) create additional inter­actions linking the chains in all directions.

## 4. Database survey

A search of the CSD (version 2024.2.0; Groom *et al.*, 2016 ▸) demonstrates that there are relatively few structurally characterized di­alkyl­dithio­phosphinates and no existing examples of structures with di­thio­phosphinate groups linked by an alkyl chain.

A number of structures contain di­phenyl­dithio­phosphinates as bidentate ligands coordinated to late transition metals such as platinum and palladium (Alison & Stephenson, 1971 ▸; Fackler *et al.*, 1982 ▸; Landtiser *et al.*, 1995 ▸). In some cases, the di­alkyl­dithio­phosphinate ends up serving as a counter-ion instead of coordinating to the metal center (Kokina *et al.*, 2008 ▸). Di­ethyl­dithio­phosphinates have also been used to form molybdenum(IV)sulfur clusters, Mo_3_S_4_(Et_2_PS_2_)_4_ (Keck *et al.*, 1981 ▸).

The structure most closely related to the title compound is that of sodium di­ethyl­dithio­phosphinate dihydrate (CSD refcode SAGWUS; Svensson & Albertsson, 1989 ▸). Each sulfur atom is hydrogen bonded to two water mol­ecules, forming an extended network in the *ab* plane, with successive layers separated by sodium cations. The sodium ions are found in a similar distorted octa­hedral environment, but with two of the six coordination sites occupied by sulfur instead of water. The octa­hedra are linked by edge sharing within the layer with every third octa­hedron missing.

## 5. Synthesis and crystallization

The title mol­ecule was prepared serendipitously while attempt­ing to remove the Cu^I^ template of a[Cu(P_2_)_2_]^+^ complex. 1,2-Bis(meth­oxy­propyl)phosphino­ethane (MeOPrPE) (2 eq.) was reacted with Cu(MeCN)_4_PF_6_ (1 eq.) in aceto­nitrile to yield the corresponding Cu(MeOPrPE)_2_PF_6_ complex. As a proof-of-concept, an attempt to remove the copper and yield the free phosphine back was performed. The copper complex was dissolved in 10 mL of THF and added to a solution of 10 eq. NaSH-hydrate in 30 mL of absolute EtOH. The mixture was refluxed for 24 h, forming Cu_2_S as a black precipitate. The reaction mixture was cooled to RT, filtered through a celite plug, then the solvent was allowed to slowly evaporate at RT in air, yielding crystals of the title mol­ecule.

## 6. Refinement

Crystal data, data collection and structure refinement details are summarized in Table 2 ▸. All hydrogen atoms were located in the difference maps. Carbon-bonded hydrogen atoms were freely refined. For the water mol­ecules the O—H distances were restrained (SADI) to have similar distances. The displacement parameters for the water hydrogen atoms were constrained to be *U*_iso_(H) = 1.5*U*_eq_(O). The occupancy of the disordered water mol­ecule (O51/O52) was fixed at 0.50 since free refinement gave an occupancy of 0.486 (8).

## Supplementary Material

Crystal structure: contains datablock(s) global, I. DOI: 10.1107/S2056989024009642/ev2010sup1.cif

Structure factors: contains datablock(s) I. DOI: 10.1107/S2056989024009642/ev2010Isup2.hkl

CCDC reference: 2388007

Additional supporting information:  crystallographic information; 3D view; checkCIF report

## Figures and Tables

**Table 1:** Hydrogen-bond geometry (Å, °)

*D*—H⋯*A*	*D*—H	H⋯*A*	*D*⋯*A*	*D*—H⋯*A*
O2—H2*C*⋯S1^i^	0.78 (1)	2.52 (1)	3.2920 (13)	167 (2)
O52—H52⋯S1^i^	0.79 (2)	2.42 (2)	3.193 (3)	167 (4)
O3—H3*AA*⋯S2^ii^	0.79 (1)	2.50 (1)	3.2840 (13)	177 (2)
O4—H4*C*⋯S2^iii^	0.78 (1)	2.49 (2)	3.2535 (14)	169 (2)
O51—H51⋯S2^iv^	0.78 (1)	2.52 (1)	3.117 (2)	134 (2)
O52—H51⋯S2^iv^	0.83 (1)	2.52 (1)	3.314 (2)	162 (2)
O2—H2*D*⋯O4^v^	0.77 (1)	2.05 (1)	2.8069 (19)	170 (2)
O3—H3*BB*⋯O3^ii^	0.79 (2)	2.02 (2)	2.801 (2)	171 (4)
O3—H3*C*⋯O52^vi^	0.71 (4)	2.04 (4)	2.748 (3)	174 (5)
O4—H4*D*⋯O51^vii^	0.75 (1)	2.07 (2)	2.731 (3)	147 (3)
O51—H51*A*⋯O3^vi^	0.79 (2)	2.05 (2)	2.825 (3)	173 (5)

Symmetry codes: (i) 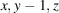; (ii) 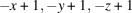; (iii) 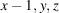; (iv) 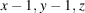; (v) 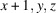; (vi) 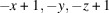; (vii) 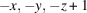.

**Table 2:** Experimental details

Crystal data	
Chemical formula	[Na_2_(C_10_H_22_O_2_P_2_S_4_)(H_2_O)_8_]
*M*_r_	277.28
Crystal system, space group	Triclinic, *P* 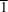
Temperature (K)	173
*a*, *b*, *c* (Å)	6.7412 (8), 8.2961 (8), 11.8621 (17)
α, β, γ (°)	79.608 (2), 89.207 (2), 87.030 (1)
*V* (Å^3^)	651.63 (14)
*Z*	2
Radiation type	Mo *K*α
μ (mm^−1^)	0.56
Crystal size (mm)	0.19 × 0.12 × 0.06

Data collection	
Diffractometer	Bruker *SMARTAPEX* CCD area detector
Absorption correction	Multi-scan (*SADABS*; Krause *et al.*, 2015 ▸)
*T*_min_, *T*_max_	0.838, 1.000
No. of measured, independent and observed [*I* > 2σ(*I*)] reflections	7596, 3007, 2736
*R*_int_	0.014
(sin θ/λ)_max_ (Å^−1^)	0.666

Refinement	
*R*[*F*^2^ > 2σ(*F*^2^)], *wR*(*F*^2^), *S*	0.026, 0.068, 1.02
No. of reflections	3007
No. of parameters	210
No. of restraints	45
H-atom treatment	H atoms treated by a mixture of independent and constrained refinement
Δρ_max_, Δρ_min_ (e Å^−3^)	0.43, −0.20

Computer programs: *SMART* and *SAINT* (Bruker, 2003 ▸), *SHELXTL* (Sheldrick, 2008 ▸), *SHELXL2018/3* (Sheldrick, 2015 ▸), *Mercury* (Macrae *et al.*, 2020 ▸), *OLEX2* (Dolomanov *et al.*, 2009 ▸) and *publCIF* (Westrip, 2010 ▸).

## supplementary crystallographic information

### catena-Poly[[triaquasodium]-di-µ-aqua-[triaquasodium]-µ-(ethane-1,2-diyl)bis[(3-methoxypropyl)phosphinodithiolato]] Crystal data

**Table:**

[Na_2_(C_10_H_22_O_2_P_2_S_4_)(H_2_O)_8_]	*Z* = 2
*M**_r_* = 277.28	*F*(000) = 294
Triclinic, *P*1	*D*_x_ = 1.413 Mg m^−^^3^
*a* = 6.7412 (8) Å	Mo *K*α radiation, λ = 0.71073 Å
*b* = 8.2961 (8) Å	Cell parameters from 4713 reflections
*c* = 11.8621 (17) Å	θ = 2.5–28.3°
α = 79.608 (2)°	µ = 0.56 mm^−^^1^
β = 89.207 (2)°	*T* = 173 K
γ = 87.030 (1)°	Block, colorless
*V* = 651.63 (14) Å^3^	0.19 × 0.12 × 0.06 mm

### catena-Poly[[triaquasodium]-di-µ-aqua-[triaquasodium]-µ-(ethane-1,2-diyl)bis[(3-methoxypropyl)phosphinodithiolato]] Data collection

**Table:**

Bruker SMART APEX CCD area detector diffractometer	3007 independent reflections
Radiation source: sealed tube	2736 reflections with *I* > 2σ(*I*)
Graphite monochromator	*R*_int_ = 0.014
phi and ω scans	θ_max_ = 28.3°, θ_min_ = 2.5°
Absorption correction: multi-scan (SADABS; Krause *et al.*, 2015)	*h* = −8→8
*T*_min_ = 0.838, *T*_max_ = 1.000	*k* = −10→10
7596 measured reflections	*l* = −15→15

### catena-Poly[[triaquasodium]-di-µ-aqua-[triaquasodium]-µ-(ethane-1,2-diyl)bis[(3-methoxypropyl)phosphinodithiolato]] Refinement

**Table:**

Refinement on *F*^2^	Primary atom site location: dual
Least-squares matrix: full	Secondary atom site location: difference Fourier map
*R*[*F*^2^ > 2σ(*F*^2^)] = 0.026	Hydrogen site location: difference Fourier map
*wR*(*F*^2^) = 0.068	H atoms treated by a mixture of independent and constrained refinement
*S* = 1.02	*w* = 1/[σ^2^(*F*_o_^2^) + (0.0357*P*)^2^ + 0.2159*P*] where *P* = (*F*_o_^2^ + 2*F*_c_^2^)/3
3007 reflections	(Δ/σ)_max_ = 0.001
210 parameters	Δρ_max_ = 0.43 e Å^−^^3^
45 restraints	Δρ_min_ = −0.20 e Å^−^^3^

### catena-Poly[[triaquasodium]-di-µ-aqua-[triaquasodium]-µ-(ethane-1,2-diyl)bis[(3-methoxypropyl)phosphinodithiolato]] Special details

**Table:**

Geometry. All esds (except the esd in the dihedral angle between two l.s. planes) are estimated using the full covariance matrix. The cell esds are taken into account individually in the estimation of esds in distances, angles and torsion angles; correlations between esds in cell parameters are only used when they are defined by crystal symmetry. An approximate (isotropic) treatment of cell esds is used for estimating esds involving l.s. planes.

### catena-Poly[[triaquasodium]-di-µ-aqua-[triaquasodium]-µ-(ethane-1,2-diyl)bis[(3-methoxypropyl)phosphinodithiolato]] Fractional atomic coordinates and isotropic or equivalent isotropic displacement parameters (Å^2^)

**Table:**

	*x*	*y*	*z*	*U*_iso_*/*U*_eq_	Occ. (<1)
S1	0.65743 (5)	0.71747 (4)	0.84910 (3)	0.02676 (10)	
S2	0.95943 (5)	0.52147 (4)	0.67995 (3)	0.02722 (10)	
P1	0.83354 (5)	0.52284 (4)	0.83414 (3)	0.01867 (9)	
C1	0.1587 (3)	−0.0034 (2)	0.84426 (17)	0.0374 (4)	
H1A	0.050 (3)	−0.019 (2)	0.7965 (17)	0.045 (5)*	
H1B	0.234 (3)	−0.108 (3)	0.8699 (17)	0.050 (6)*	
H1C	0.101 (3)	0.035 (3)	0.906 (2)	0.060 (7)*	
C2	0.4380 (2)	0.15096 (17)	0.85447 (12)	0.0257 (3)	
H2A	0.529 (3)	0.058 (2)	0.8688 (15)	0.032 (4)*	
H2B	0.381 (3)	0.164 (2)	0.9249 (16)	0.032 (4)*	
C3	0.5383 (2)	0.30505 (17)	0.79961 (12)	0.0254 (3)	
H3A	0.589 (3)	0.293 (2)	0.7283 (16)	0.034 (5)*	
H3B	0.443 (3)	0.396 (2)	0.7870 (15)	0.032 (4)*	
C4	0.7036 (2)	0.33531 (17)	0.87807 (12)	0.0258 (3)	
H4A	0.650 (3)	0.341 (2)	0.9483 (16)	0.035 (5)*	
H4B	0.801 (3)	0.250 (2)	0.8855 (16)	0.041 (5)*	
C5	1.0347 (2)	0.50194 (19)	0.93811 (11)	0.0243 (3)	
H5A	1.110 (3)	0.409 (2)	0.9306 (15)	0.035 (5)*	
H5B	1.112 (3)	0.591 (2)	0.9135 (15)	0.033 (5)*	
Na1	0.33236 (8)	0.08688 (6)	0.58806 (5)	0.02448 (13)	
O1	0.28216 (15)	0.11749 (12)	0.78382 (8)	0.0281 (2)	
O2	0.66857 (17)	−0.02101 (15)	0.60507 (10)	0.0331 (3)	
H2C	0.683 (3)	−0.089 (2)	0.6599 (14)	0.050*	
H2D	0.754 (3)	0.037 (2)	0.6050 (18)	0.050*	
O3	0.42969 (18)	0.35106 (14)	0.48142 (10)	0.0309 (2)	
H3AA	0.339 (3)	0.382 (2)	0.4406 (16)	0.046*	
H3BB	0.480 (6)	0.432 (3)	0.488 (4)	0.046*	0.5
H3C	0.501 (7)	0.313 (5)	0.448 (4)	0.046*	0.5
O4	−0.00709 (18)	0.18169 (19)	0.57874 (12)	0.0445 (3)	
H4C	−0.020 (4)	0.255 (2)	0.6113 (19)	0.067*	
H4D	−0.047 (4)	0.215 (3)	0.5202 (15)	0.067*	
O51	0.2410 (4)	−0.1906 (3)	0.6086 (2)	0.0316 (5)	0.5
H51	0.209 (3)	−0.259 (2)	0.6588 (15)	0.047*	
H51A	0.332 (5)	−0.242 (5)	0.589 (4)	0.047*	0.5
O52	0.3145 (4)	−0.2131 (3)	0.6610 (2)	0.0279 (5)	0.5
H52	0.391 (5)	−0.245 (5)	0.711 (3)	0.042*	0.5

### catena-Poly[[triaquasodium]-di-µ-aqua-[triaquasodium]-µ-(ethane-1,2-diyl)bis[(3-methoxypropyl)phosphinodithiolato]] Atomic displacement parameters (Å^2^)

**Table:**

	*U*^11^	*U*^22^	*U*^33^	*U*^12^	*U*^13^	*U*^23^
S1	0.0338 (2)	0.02347 (17)	0.02226 (18)	0.00774 (14)	−0.00349 (14)	−0.00436 (13)
S2	0.03110 (19)	0.02942 (19)	0.01979 (17)	0.00183 (14)	0.00272 (13)	−0.00193 (13)
P1	0.02118 (17)	0.01825 (16)	0.01566 (16)	−0.00006 (12)	−0.00169 (12)	−0.00069 (12)
C1	0.0294 (8)	0.0350 (9)	0.0437 (10)	−0.0088 (7)	0.0076 (7)	0.0057 (7)
C2	0.0362 (8)	0.0205 (7)	0.0205 (7)	−0.0040 (6)	0.0011 (6)	−0.0036 (5)
C3	0.0343 (8)	0.0221 (7)	0.0195 (7)	−0.0055 (6)	−0.0003 (6)	−0.0017 (5)
C4	0.0368 (8)	0.0207 (7)	0.0194 (7)	−0.0054 (6)	−0.0027 (6)	−0.0007 (5)
C5	0.0223 (7)	0.0291 (7)	0.0199 (7)	0.0009 (6)	−0.0020 (5)	−0.0009 (5)
Na1	0.0237 (3)	0.0255 (3)	0.0249 (3)	−0.0031 (2)	−0.0013 (2)	−0.0055 (2)
O1	0.0341 (6)	0.0263 (5)	0.0237 (5)	−0.0114 (4)	0.0028 (4)	−0.0017 (4)
O2	0.0330 (6)	0.0364 (6)	0.0307 (6)	0.0025 (5)	−0.0107 (5)	−0.0089 (5)
O3	0.0313 (6)	0.0240 (5)	0.0379 (7)	−0.0031 (4)	−0.0060 (5)	−0.0061 (5)
O4	0.0266 (6)	0.0632 (9)	0.0524 (8)	0.0012 (6)	−0.0069 (5)	−0.0341 (7)
O51	0.0327 (13)	0.0287 (12)	0.0326 (13)	−0.0039 (10)	0.0035 (11)	−0.0027 (10)
O52	0.0267 (12)	0.0293 (12)	0.0274 (13)	−0.0076 (9)	−0.0047 (10)	−0.0017 (10)

### catena-Poly[[triaquasodium]-di-µ-aqua-[triaquasodium]-µ-(ethane-1,2-diyl)bis[(3-methoxypropyl)phosphinodithiolato]] Geometric parameters (Å, º)

**Table:**

S1—P1	1.9867 (5)	Na1—Na1^ii^	3.4885 (11)
S2—P1	2.0073 (5)	Na1—O1	2.3986 (12)
P1—C4	1.8155 (14)	Na1—O2^ii^	2.4494 (13)
P1—C5	1.8262 (14)	Na1—O2	2.3908 (13)
C1—H1A	0.96 (2)	Na1—O3	2.4413 (13)
C1—H1B	0.99 (2)	Na1—H3C	2.57 (4)
C1—H1C	0.93 (2)	Na1—O4	2.3783 (13)
C1—O1	1.4219 (18)	Na1—O51	2.383 (2)
C2—H2A	0.951 (18)	Na1—O52	2.491 (2)
C2—H2B	0.937 (18)	O2—H2C	0.783 (13)
C2—C3	1.5141 (19)	O2—H2D	0.768 (14)
C2—O1	1.4218 (18)	O3—H3AA	0.788 (14)
C3—H3A	0.929 (19)	O3—H3BB	0.786 (16)
C3—H3B	0.959 (18)	O3—H3C	0.71 (4)
C3—C4	1.521 (2)	O4—H4C	0.775 (14)
C4—H4A	0.911 (19)	O4—H4D	0.748 (14)
C4—H4B	0.93 (2)	O51—H51	0.782 (14)
C5—C5^i^	1.530 (3)	O51—H51A	0.785 (16)
C5—H5A	0.918 (19)	O52—H51	0.826 (14)
C5—H5B	0.928 (18)	O52—H52	0.789 (16)
			
S1—P1—S2	116.51 (2)	O2—Na1—O3	92.52 (4)
C4—P1—S1	110.54 (6)	O2—Na1—H3C	80.5 (10)
C4—P1—S2	109.33 (5)	O2^ii^—Na1—H3C	70.7 (10)
C4—P1—C5	102.98 (7)	O2—Na1—O52	74.03 (7)
C5—P1—S1	109.49 (5)	O2^ii^—Na1—O52	86.90 (7)
C5—P1—S2	107.08 (5)	O3—Na1—Na1^ii^	85.84 (4)
H1A—C1—H1B	110.3 (16)	O3—Na1—O2^ii^	81.58 (4)
H1A—C1—H1C	105.4 (18)	O3—Na1—H3C	16.1 (10)
H1B—C1—H1C	110.3 (18)	O3—Na1—O52	162.66 (7)
O1—C1—H1A	109.7 (12)	O4—Na1—Na1^ii^	136.49 (4)
O1—C1—H1B	111.5 (12)	O4—Na1—O1	80.67 (4)
O1—C1—H1C	109.5 (14)	O4—Na1—O2^ii^	93.34 (5)
H2A—C2—H2B	107.4 (14)	O4—Na1—O2	176.86 (6)
C3—C2—H2A	111.9 (11)	O4—Na1—O3	90.54 (5)
C3—C2—H2B	110.5 (10)	O4—Na1—H3C	102.6 (10)
O1—C2—H2A	109.2 (10)	O4—Na1—O51	91.08 (8)
O1—C2—H2B	107.1 (11)	O4—Na1—O52	103.10 (7)
O1—C2—C3	110.62 (11)	O51—Na1—Na1^ii^	75.92 (6)
C2—C3—H3A	109.8 (11)	O51—Na1—O1	97.55 (7)
C2—C3—H3B	109.9 (10)	O51—Na1—O2^ii^	73.40 (7)
C2—C3—C4	108.62 (12)	O51—Na1—O2	86.43 (7)
H3A—C3—H3B	107.0 (14)	O51—Na1—O3	154.98 (8)
C4—C3—H3A	110.6 (11)	O52—Na1—H3C	146.6 (10)
C4—C3—H3B	110.9 (10)	C1—O1—Na1	112.06 (10)
P1—C4—H4A	106.5 (11)	C2—O1—C1	111.26 (12)
P1—C4—H4B	105.7 (12)	C2—O1—Na1	122.95 (9)
C3—C4—P1	116.31 (10)	Na1—O2—Na1^ii^	92.22 (4)
C3—C4—H4A	108.2 (11)	Na1—O2—H2C	111.4 (16)
C3—C4—H4B	110.7 (12)	Na1^ii^—O2—H2C	122.1 (16)
H4A—C4—H4B	109.2 (16)	Na1—O2—H2D	120.1 (16)
P1—C5—H5A	107.2 (11)	Na1^ii^—O2—H2D	105.4 (16)
P1—C5—H5B	105.3 (11)	H2C—O2—H2D	106 (2)
C5^i^—C5—P1	114.30 (13)	Na1—O3—H3AA	104.3 (15)
C5^i^—C5—H5A	111.7 (11)	Na1—O3—H3BB	143 (3)
C5^i^—C5—H5B	110.9 (11)	Na1—O3—H3C	92 (4)
H5A—C5—H5B	107.1 (15)	H3AA—O3—H3BB	104 (3)
Na1^ii^—Na1—H3C	69.8 (10)	H3AA—O3—H3C	106 (4)
O1—Na1—Na1^ii^	141.45 (4)	Na1—O4—H4C	108.4 (19)
O1—Na1—O2^ii^	169.16 (4)	Na1—O4—H4D	116 (2)
O1—Na1—O3	107.34 (4)	H4C—O4—H4D	104 (3)
O1—Na1—H3C	119.4 (10)	Na1—O51—H51	136.6 (15)
O1—Na1—O52	85.69 (6)	Na1—O51—H51A	108 (3)
O2^ii^—Na1—Na1^ii^	43.22 (3)	H51—O51—H51A	95 (3)
O2—Na1—Na1^ii^	44.56 (3)	Na1—O52—H51	120.9 (13)
O2—Na1—O1	97.76 (4)	Na1—O52—H52	112 (3)
O2—Na1—O2^ii^	87.78 (4)	H51—O52—H52	120 (3)
			
S1—P1—C4—C3	69.75 (12)	C3—C2—O1—C1	−166.90 (13)
S1—P1—C5—C5^i^	53.71 (16)	C3—C2—O1—Na1	56.02 (14)
S2—P1—C4—C3	−59.78 (13)	C4—P1—C5—C5^i^	−63.91 (16)
S2—P1—C5—C5^i^	−179.14 (13)	C5—P1—C4—C3	−173.38 (12)
C2—C3—C4—P1	−175.75 (10)	O1—C2—C3—C4	−179.89 (12)

Symmetry codes: (i) −*x*+2, −*y*+1, −*z*+2; (ii) −*x*+1, −*y*, −*z*+1.

### catena-Poly[[triaquasodium]-di-µ-aqua-[triaquasodium]-µ-(ethane-1,2-diyl)bis[(3-methoxypropyl)phosphinodithiolato]] Hydrogen-bond geometry (Å, º)

**Table:**

*D*—H···*A*	*D*—H	H···*A*	*D*···*A*	*D*—H···*A*
O2—H2*C*···S1^iii^	0.78 (1)	2.52 (1)	3.2920 (13)	167 (2)
O52—H52···S1^iii^	0.79 (2)	2.42 (2)	3.193 (3)	167 (4)
O3—H3*AA*···S2^iv^	0.79 (1)	2.50 (1)	3.2840 (13)	177 (2)
O4—H4*C*···S2^v^	0.78 (1)	2.49 (2)	3.2535 (14)	169 (2)
O51—H51···S2^vi^	0.78 (1)	2.52 (1)	3.117 (2)	134 (2)
O52—H51···S2^vi^	0.83 (1)	2.52 (1)	3.314 (2)	162 (2)
O2—H2*D*···O4^vii^	0.77 (1)	2.05 (1)	2.8069 (19)	170 (2)
O3—H3*BB*···O3^iv^	0.79 (2)	2.02 (2)	2.801 (2)	171 (4)
O3—H3*C*···O52^ii^	0.71 (4)	2.04 (4)	2.748 (3)	174 (5)
O4—H4*D*···O51^viii^	0.75 (1)	2.07 (2)	2.731 (3)	147 (3)
O51—H51*A*···O3^ii^	0.79 (2)	2.05 (2)	2.825 (3)	173 (5)

Symmetry codes: (ii) −*x*+1, −*y*, −*z*+1; (iii) *x*, *y*−1, *z*; (iv) −*x*+1, −*y*+1, −*z*+1; (v) *x*−1, *y*, *z*; (vi) *x*−1, *y*−1, *z*; (vii) *x*+1, *y*, *z*; (viii) −*x*, −*y*, −*z*+1.
